# A Genetically Encoded Marker for Imaging of Internal *Plasmodium* Membranes

**DOI:** 10.1111/mmi.70098

**Published:** 2026-09-15

**Authors:** Leah Zink, Viola Reuschenbach, Samy Sid Ahmed, Muriel Lauer, Jana Niethammer, Aistė Kudulytė, Katharina Zorko, Oliver T. Fackler, Franziska Hentzschel, Markus Ganter

**Affiliations:** ^1^ Center for Infectious Diseases, Parasitology, Medical Faculty Heidelberg Heidelberg University Heidelberg Germany; ^2^ Center for Infectious Diseases, Integrative Virology, Medical Faculty Heidelberg Heidelberg University Heidelberg Germany; ^3^ German Centre for Infection Research (DZIF) Partner Site Heidelberg Heidelberg Germany

**Keywords:** internal membrane marker, live‐cell imaging, *Plasmodium* spp.

## Abstract

Fluorescence microscopy is a powerful tool to analyze the subcellular architecture, and long‐term live‐cell imaging permits the analysis of the dynamics of intracellular structures, such as membranes. Fluorescent labeling of the plasma membrane of *Plasmodium*, the causative agent of malaria, via membrane‐resident proteins has been described, but proteins that broadly mark internal membranes are currently elusive. Alternatively, general membrane dyes can be employed to study membrane dynamics; however, we find that the membrane dye BODIPY TR Ceramide has adverse effects on cell viability during live‐cell imaging. To overcome this limitation, we present IMMP (Internal Membrane Marker for *Plasmodium*), a 32‐amino acid long peptide derived from *P. falciparum* PCNA1, which targets (fluorescent) protein tags to internal membranes for detection in live or fixed samples. Importantly, IMMP enables non‐invasive, long‐term live‐cell imaging of internal membranes without affecting *P. falciparum* viability and is functional in blood stages of both the human malaria parasite *P. falciparum* and the rodent‐infecting parasite *P. berghei*, as well as in *P. berghei* mosquito stages. IMMP is also compatible with advanced techniques such as expansion microscopy. Together, our results establish IMMP as a tool for internal membrane imaging in *Plasmodium*.

## Graphical abstract

IMMP is a peptide‐based marker that labels internal membranes across multiple stages and species of the malaria‐causing parasite *Plasmodium*. It works in both live and fixed samples and supports long‐term live‐cell imaging.

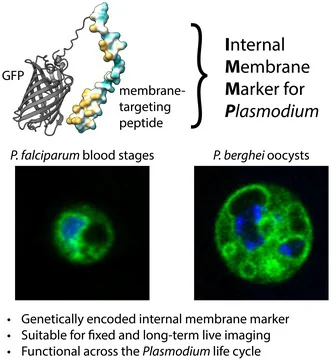

## 1 Introduction

Malaria remains a major public health burden with an estimated 282 million cases in 2024. Of the over 600,000 malaria‐related deaths in the same year, most were children under the age of five in Sub‐Saharan Africa (World Malaria Report 2025). Malaria is caused by unicellular eukaryotes of the genus *Plasmodium*, with at least five species infecting humans. *P. falciparum* is responsible for the most virulent form of human malaria, which also accounts for the vast majority of cases and fatalities (World Malaria Report 2025). To ultimately tackle malaria, a better understanding of the basic biology of *Plasmodium* spp. is needed.

A powerful tool to study *Plasmodium* spp. biology is fluorescence microscopy, which offers insight into the parasites' subcellular architecture. Long‐term live‐cell imaging permits the analysis of the dynamics of internal structures such as organelles and membranes. For this, detectable marker proteins or specific dyes can be employed.

To visualize the parasite plasma membrane (PPM) in the rodent‐infecting *P. berghei*, a reporter system based on the Plasma Membrane Protein 1 (PBANKA_0809400) was previously established (Burda et al. 2017). Similarly, the PPM of the human pathogen *P. falciparum* can be visualized, for example, by endogenous tagging of the plasma membrane resident transporter 1 (PMRT1) (Wichers et al. 2022). However, similar genetically encoded fluorescent markers are currently lacking for the labeling of internal parasite membranes.

To mark all membranes independent of the expression dynamics and localization of a given reporter, commercially available BODIPY Ceramide dyes are commonly used. They combine the fluorescence properties of BODIPY with the sphingolipid ceramide, which supports membrane localization (Karolin et al. 1994; Marks et al. 2008). These dyes have been used to stain lipids in different *Plasmodium* species for live‐cell imaging as well as fixed‐cell imaging (Grüring et al. 2011; Liffner and Absalon 2021; Liffner et al. 2023).

Despite the advantage of being a readily applicable and robust lipid dye, BODIPY Ceramide‐associated phototoxicity during live‐cell imaging was previously described (Kähärä et al. 2022), which is likely due to the formation of highly reactive singlet oxygen species close to the membrane (Liang et al. 2020). Thus, in particular for long‐term live‐cell imaging of *Plasmodium* spp., an alternative membrane marker may be useful.

To investigate the nucleocytoplasmic transport of *P. falciparum* PCNA1 (PF3D7_1361900), which transiently accumulates in a subset of nuclei of a *P. falciparum* schizont (Klaus et al. 2022), we predicted possible nuclear localization signals (NLS) and nuclear export signals (NES) in PCNA1 (Kosugi et al. 2009; Xu et al. 2015). To assess whether these predicted signals are sufficient for nuclear or cytoplasmic targeting, we fused them to GFP. We serendipitously found that a 32‐amino acid predicted nuclear localization signal of *P. falciparum* PCNA1 targets protein tags to membranes, and we developed this peptide into a membrane marker for imaging.

## 2 Results

To investigate the dynamics of *P. falciparum* proliferation inside human red blood cells (RBCs), we developed fluorescent reporter cell lines, which permit, for example, the tracking of DNA replication and nuclear division events in the schizont stage over time (Klaus et al. 2022). These reporter cell lines can be combined with fluorescent dyes to investigate the dynamics of additional cellular structures over time (Machado et al. 2023). Yet, when we paired fluorescent reporter lines with the lipid dye BODIPY TR Ceramide (Marks et al. 2008), we noticed a marked susceptibility of the cells to illumination during imaging. To quantify this effect, we compared the survival of uninfected and *P. falciparum*‐infected RBCs during imaging with and without BODIPY TR Ceramide staining (Figure 1).

**FIGURE 1:**
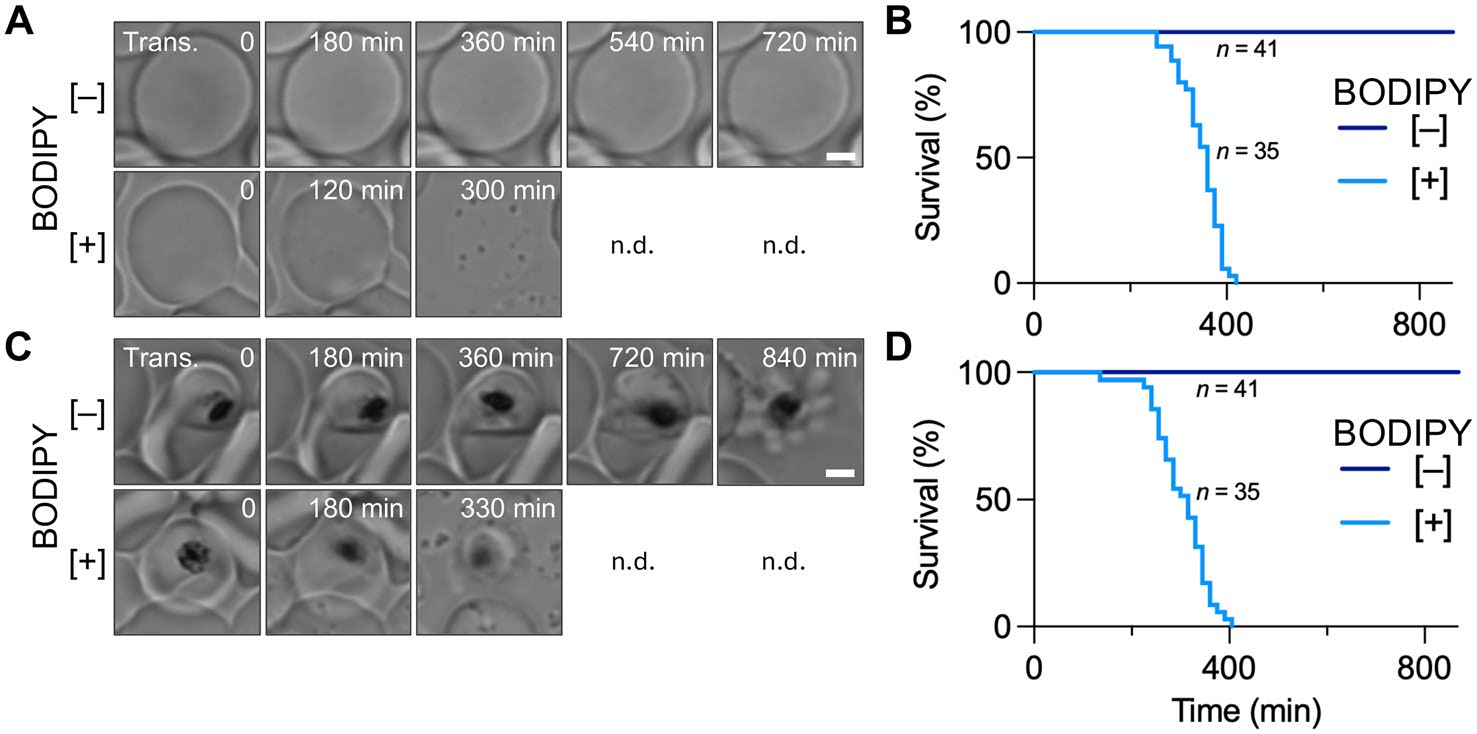
BODIPY TR Ceramide staining affects RBC survival during live‐cell imaging. (A) Time‐lapse microscopy of RBCs, unstained [−] and stained [+] with BODIPY TR Ceramide. (B) Survival of RBCs during imaging. (C) Time‐lapse microscopy of RBCs infected with *P. falciparum*, unstained [−] and stained [+] with BODIPY TR Ceramide. (D) Survival of RBCs infected with *P. falciparum* during imaging. Scale bars, 2 μm.

Using relatively gentle imaging conditions (i.e., 0.2% laser power for BODIPY TR Ceramide and 0.1% for transmission image detection, z‐stack with 11 slices every 15 min), we found that none of the unstained cells demised, regardless of whether they were *P. falciparum*‐infected or not. In contrast, BODIPY TR Ceramide‐stained cells were all lysed after approx. 400 min of imaging, with a slightly earlier onset of lysis in infected cells. Increased laser power during imaging led to a much earlier RBC lysis in both uninfected and infected cells (Figure S1), supporting previous reports of phototoxicity associated with BODIPY‐based dyes (Kähärä et al. 2022).

With this observation in mind, we serendipitously noticed that a 32‐amino acid peptide derived from *P. falciparum* PCNA1 (33 amino acids including the methionine from the start codon) appeared to target GFP to membranes in fixed cells (Figure 2A,B). Previously, we have shown that PCNA1 transiently accumulates in the subset of nuclei of a multinucleated schizont that are replicating their DNA (Klaus et al. 2022; Machado et al. 2023). To investigate its nucleocytoplasmic transport, we fused multiple predicted NLS and NES of PCNA1 to GFP via a flexible (GGGGS)_2_ linker (Chen et al. 2013). These fusion proteins were episomally expressed under the control of the *P. falciparum* CRT promoter and the *P. berghei* DHFR/TS terminator. Assessing their subcellular localization, we found that the predicted NLS1::GFP fusion did not localize to the nucleus but appeared to localize to internal membranes (Figure 2A,B). Given the phototoxic effects of BODIPY TR Ceramide staining, especially during long‐term live‐cell imaging (Figure 1 and Figure S1), we assessed whether this 32‐amino acid peptide can serve as an alternative Internal Membrane Marker for *Plasmodium* (IMMP) (Figure 2A,B).

**FIGURE 2:**
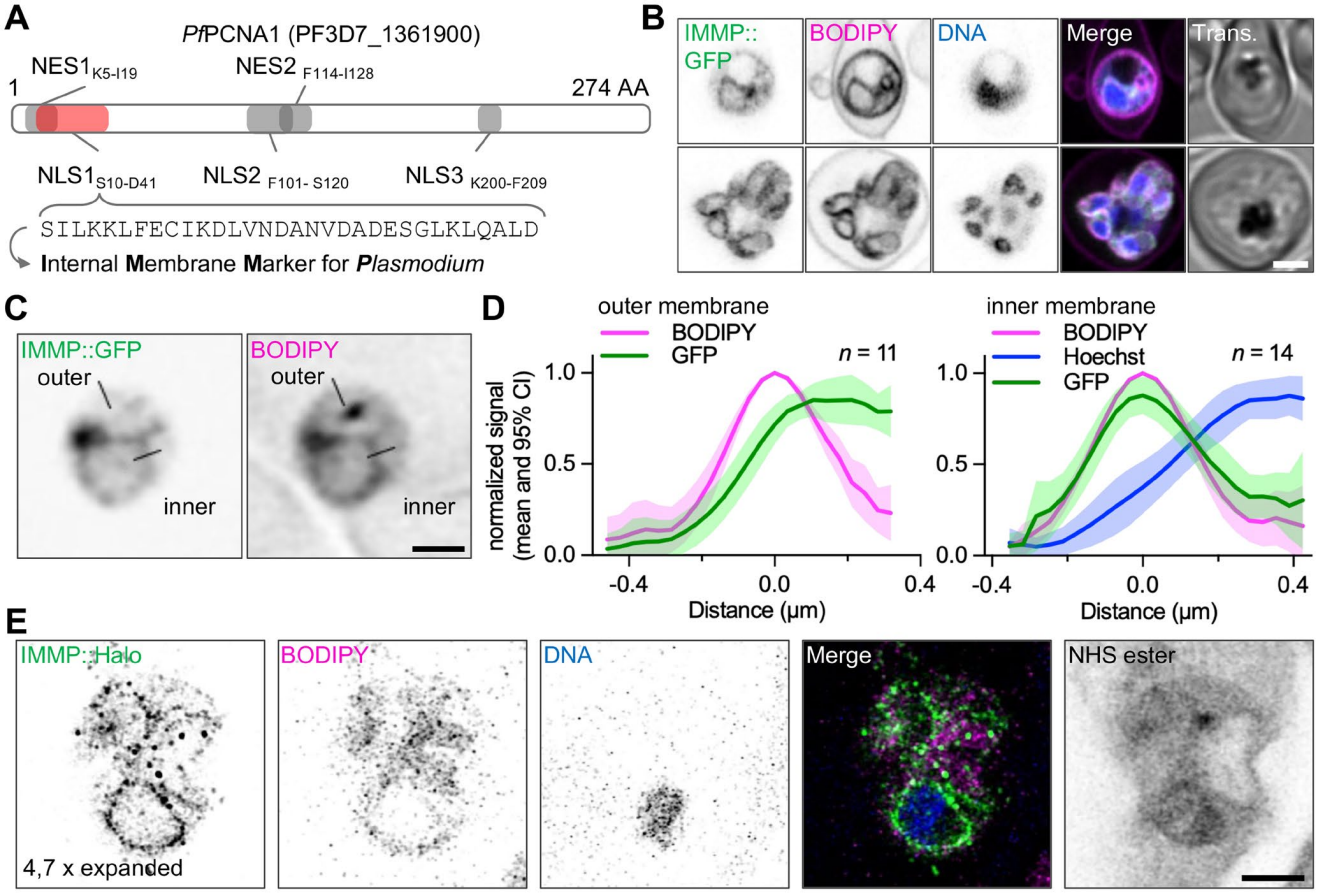
A 32‐amino acid peptide targets proteins to internal membranes in fixed cells. (A) Illustration of the position of predicted nuclear localization (NLS) and nuclear export signals (NES) in the sequence of *Pf*PCNA1. The primary sequence of the 32‐amino acid long predicted NLS1 is shown below, which constitutes the Internal Membrane Marker for *Plasmodium*, IMMP. (B) In chemically fixed cells, fusion of IMMP to GFP showed a signal proximal to membranes, similar to the membrane marker BODIPY TR Ceramide. Cells were co‐stained with the DNA dye Hoechst 33342. Scale bar, 2 μm. (C) Comparison of IMMP::GFP and BODIPY TR Ceramide signal intensities in a trophozoite stage parasite. Lines highlight representative positions of line plots for outer and inner membranes; scale bar, 1 μm. (D) Quantification of IMMP::GFP and BODIPY TR Ceramide signal intensities in different developmental stages; omitting segmenter stages, where outer and inner membranes are in close proximity, as can be seen in (B). For inner membranes, the signal of the DNA dye Hoechst 33342 was also plotted. Line, mean; band, 95% CI; min‐max normalized, with the maximal BODIPY TR Ceramide value set at distance 0 μm. (E) Ultrastructure expansion microscopy (U‐ExM) of a cell line expressing IMMP fused to a Halo‐tag and detected by an anti‐Halo antibody; co‐stained with BODIPY TR Ceramide and the DNA dye Hoechst 33342; scale bar, 4 μm.

Structure prediction of IMMP::GFP employing AlphaFold 3 (Abramson et al. 2024) indicated a short α‐helical structure followed by a disordered region, which stretches over the linker peptide into the structured GFP (Figure S2A). We noted that the IMMP alpha helix was longitudinally split into a hydrophilic and hydrophobic side (Figure S2B), potentially resulting in an amphiphilic peptide. Notably, the apparent membrane targeting was not observed when expressing GFP alone (Figure S2C). We also tested if IMMP resembles a signal peptide, which would target the fusion protein to the ER and the secretory pathway. Using the online peptide prediction tool SignalP6 (Teufel et al. 2022), we found that the likelihood of IMMP being a functional signal peptide was very low (< 0.001%), suggesting that IMMP does not localize to membranes via the ER. This notion was further supported as treatment with the secretory pathway inhibitor brefeldin A (Benting et al. 1994) did not affect the localization of IMMP::GFP (Figure S2D).

Next, we quantified the signal intensities of IMMP::GFP and BODIPY TR Ceramide in chemically fixed *P. falciparum* cells (Figure 2C,D). For this, we used an asynchronous culture of IMMP::GFP expressing parasites, and we omitted segmenter stages, where outer and inner membranes are in close proximity, such that they cannot be differentiated with confidence. At the cell periphery, BODIPY TR Ceramide stained the outer membranes (likely plasma membrane and parasitophorous vacuole membrane), which translated in a signal increase at the periphery and a decrease of signal in the cytosol (Figure 2D, left panel). IMMP::GFP signal intensity, however, was initially low at the cell periphery and increased to a stable signal in the cytosol, as also expected for a cytosolic protein. In contrast, at inner membranes, both BODIPY TR Ceramide and IMMP::GFP signal colocalized in a single peak (Figure 2D, right panel). This suggests that IMMP targets GFP predominantly to internal membranes.

To test if IMMP permits a marking of membranes in ultrastructure expansion microscopy (U‐ExM) (Gambarotto et al. 2019; Bertiaux et al. 2021; Liffner and Absalon 2021), we fused it to a Halo tag, which we detected with an antibody. In expanded cells, IMMP::Halo showed a similar subcellular localization to internal membranes like IMMP::GFP in unexpanded cells (Figure 2E and Figure S3). Previously, BODIPY TR Ceramide has been successfully used in U‐ExM (Liffner and Absalon 2021, 2024; Liffner et al. 2023). In our hands, however, it showed an inferior signal compared to IMMP::Halo (Figure 2E and Figure S3), potentially due to signal amplification by antibody staining.

Subsequently, we assessed IMMP::GFP during live‐cell imaging. Similar to fixed cells (Figure 2B–E), short‐term live‐cell imaging revealed that IMMP::GFP localized predominantly to internal membranes, comparable to BODIPY TR Ceramide staining (Figure 3A). Notably, during long‐term live‐cell imaging, IMMP::GFP expressing parasites did not lyse, and although the GFP intensity varied from cell to cell, it remained relatively stable over 800 min of imaging (Figure 3B,C). Since IMMP::GFP was expressed from an episomal plasmid, we also tested if selection for cells with a higher plasmid copy number translated into an increased IMMP::GFP signal (Epp et al. 2008). We increased the drug selection pressure by culturing IMMP::GFP expressing parasites in a 10‐fold WR99210 concentration (i.e., 25 nM). While the overall staining pattern remained similar, this led to an approximately 25‐fold brighter GFP signal (Figure 3C), suggesting that signal intensity can be adapted to experimental needs by modulating the drug selection pressure.

**FIGURE 3:**
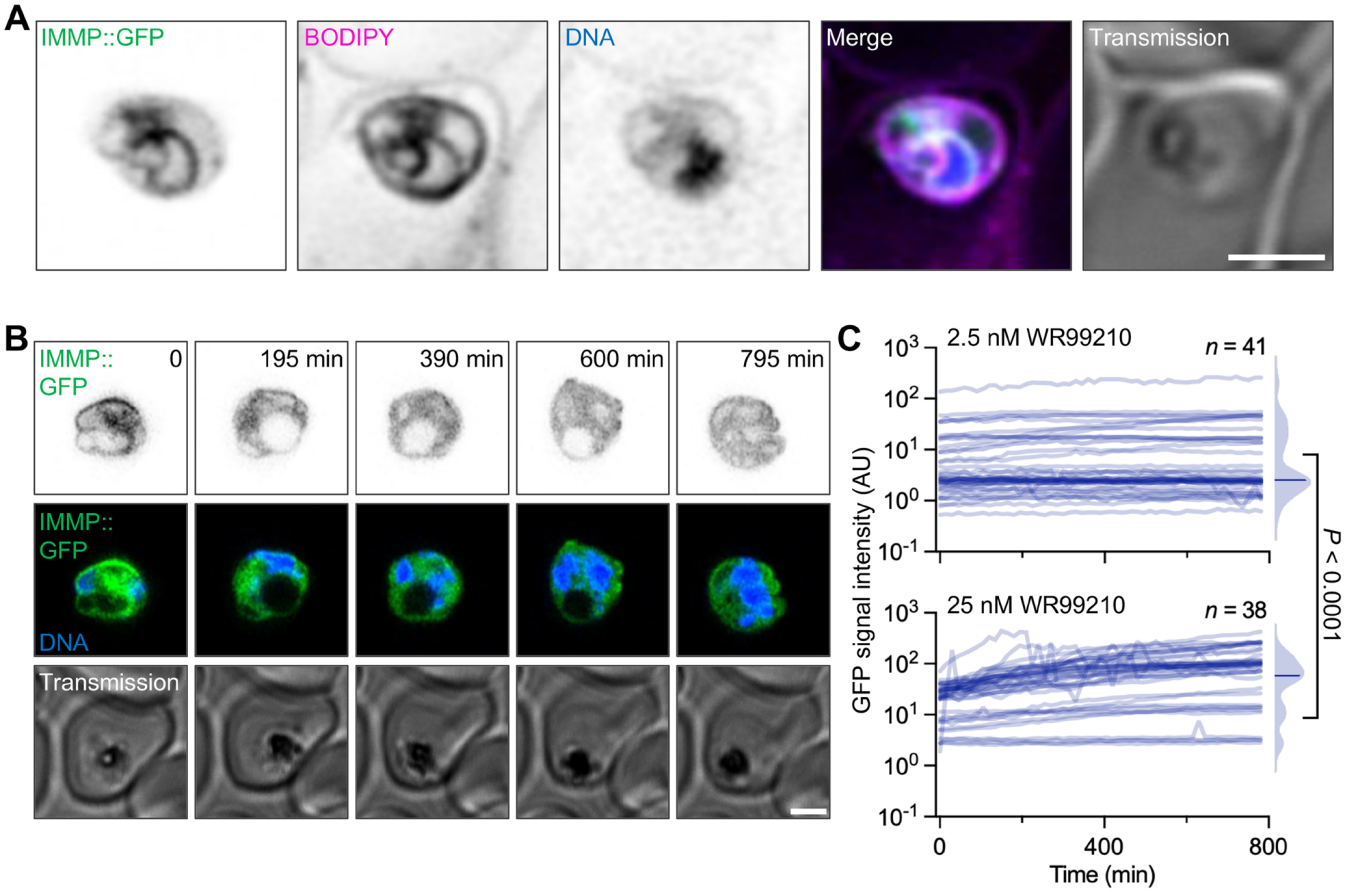
IMMP::GFP marks membranes in short‐term and long‐term live‐cell imaging. (A) Short‐term live‐cell imaging IMMP::GFP expressing parasites co‐stained with BODIPY TR Ceramide and the DNA dye SPY650‐DNA. (B) Long‐term live‐cell imaging of IMMP::GFP expressing parasites co‐stained with the DNA dye SPY650‐DNA. (C) Quantification of the IMMP::GFP signal intensity over time. Increasing the drug pressure for the presence of the IMMP::GFP expressing and WR99210‐resistance conferring plasmid selected for on average approx. 25–fold brighter parasites. Half violins, average values of individual parasites over time; line, median; *p‐*value, Mann Whitney test. Scale bars, 2 μm.

Together, this suggests that IMMP could be a substitute for BODIPY TR Ceramide staining to mark internal membranes during long‐term live‐cell imaging of *P. falciparum* blood stages.

Next, we tested if IMMP could also serve as a marker for internal membranes in other *Plasmodium* species, as well as other life cycle stages. In the rodent‐malaria parasite *P. berghei*, we integrated an IMMP::mScarlet expression construct in the silent intergenic locus on chromosome 6 (SIL6) (Kooij et al. 2012) (Figure S4A,B). Expression of IMMP::mScarlet from this endogenous locus and driven by the *hsp70* promoter led to a slight but significant reduction in parasite growth rate from an 8‐ to a 6‐fold increase over 24 h (Figure 4A and Figure S4C). Similar to *P. falciparum*, IMMP::mScarlet localized to internal membranes, also marked with BODIPY FL, in both fixed and live *P. berghei* asexual blood stages (Figure 4B).

**FIGURE 4:**
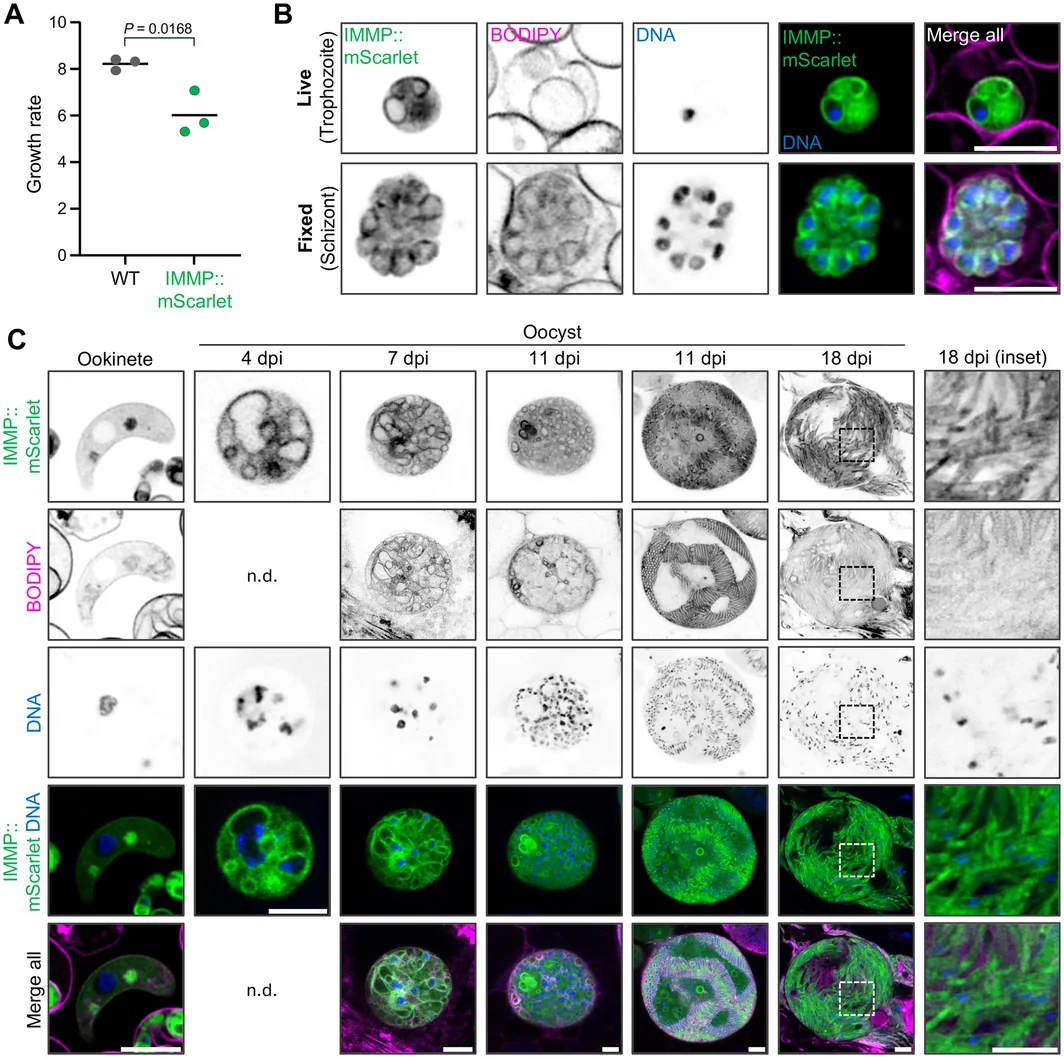
IMMP marks internal membranes throughout *P. berghei* development in the vertebrate blood and the *Anopheles* mosquito. (A) *P. berghei* parasites expressing IMMP::mScarlet endogenously from the SIL6 locus show a slightly reduced growth in asexual blood stages. Bar, mean; *p*‐value, unpaired two‐tailed *t*‐test. (B) Fixed‐ and live‐cell short‐term imaging of IMMP::mScarlet‐expressing asexual blood stage *P. berghei* parasites, co‐stained with BODIPY FL and the DNA dye Hoechst 33342. (C) Live‐cell imaging of different mosquito‐stage *P. berghei*, expressing IMMP::mScarlet and co‐stained with BODIPY FL and the DNA dye Hoechst 33342. Scale bars, 5 μm.

We then followed IMMP localization in mosquito‐infecting stages of *P. berghei*. In ookinetes, endogenously expressed IMMP::mScarlet appeared to localize predominantly to two distinct spots, possibly the crystalloids, which were also detectable with BODIPY FL (Figure 4C). The crystalloids are specific organelles rich in membranes and membrane‐associated proteins such as a palmitoyl‐*S*‐acyl transferase or the multipass transmembrane protein NAD(P) transhydrogenase (Saeed et al. 2015, 2020; Santos et al. 2016). Notably, BODIPY FL also stained ookinetes weaker than asexual blood stages. In early oocysts (Day 4 post infection), we observed an extended internal network of membranes, including the nuclear membrane and possibly the ER (Figure 4C). We noted that the nuclear compartment, as identified by IMMP::mScarlet, appeared often much larger than the signal derived from the DNA dye, potentially indicating a level of intranuclear compartmentalization in these polyploid nuclei. At later stages of oocyst development, some of the internal membrane structures seemed not to be stained with BODIPY FL, or only weakly, possibly because of difficulties of the dye to penetrate the oocyst capsule, highlighting the advantage of using a genetically encoded membrane marker over an externally added dye. In sporulating oocysts, IMMP::mScarlet additionally localized to foci adjacent to the nucleus of the sporozoites, potentially marking the nucleus associated ER (Figure 4C).

Finally, we aimed to test if IMMP was able to mark membranes in cells from evolutionary distant organisms. To this end, we transfected human HeLa‐derived TZM‐bl cells (Wei et al. 2002) with an IMMP::GFP expression construct. Fluorescence microscopy revealed a homogenously distributed signal in the cells' cytoplasm and nucleus (Figure S5A–C). This distribution was reminiscent to that of GFP alone and Western Blot analysis confirmed that the majority of ectopically expressed protein in these cells was GFP without the 32 amino acid *P. falciparum* PCNA1‐derived peptide. The lack of detection of full length IMMP::GFP in these cells may reflect, for example, protein degradation and/or the use of an alternative start codon. While more human cell types need to be tested for a generalized conclusion, this result suggests that expression of IMMP::GFP is subject to different regulation in *Plasmodium* spp. versus human cells and, thus, may serve as a *Plasmodium*‐specific membrane marker.

Together, our data indicate that IMMP can be used to track the dynamic development of internal membranes in different developmental stages of *Plasmodium*.

## 3 Discussion

We here report the serendipitous discovery of a genetically encoded membrane marker, IMMP, which allows visualization of internal membranes both in fixed and in live *Plasmodium* cells. Importantly, expression of IMMP::GFP permits long‐term live cell imaging of membrane dynamics over at least 12 h, avoiding the cytotoxicity observed with BODIPY TR Ceramide under the same conditions. Although not readily usable like dyes, IMMP marks only membranes of the parasite without contributing signal from the host cell. While the membrane localization can be attributed to the IMMP peptide [rather than the GFP (Figure S2C) or the frequently used (GGGGS)_x_ linker (Chen et al. 2013; Ambekar et al. 2022; Klaus et al. 2022)], the mechanism by which IMMP targets protein tags to membranes is unknown and remains a question for future research.

Similarly, the precise identity of the membranous structures marked by IMMP remains elusive. Modeling of its structure revealed a possibly amphiphilic alpha helix that might bind laterally to the membrane. However, the non‐uniform staining of *Plasmodium* membranes (Figure 2D) suggests a dependence on specific factors. These may be the lipid composition or specific membrane‐associated proteins. Although our initial analysis did not provide evidence for it, IMMP may resemble a non‐canonical signal peptide, which would target the fluorescent reporter proteins to the ER and from there to other internal membranes. Comparison to previously established ER‐markers will help to test if IMMP resembles a non‐canonical signal peptide (Greca et al. 1997; Dooren et al. 2005; Lee et al. 2008; Kaiser et al. 2016; Tchoufack et al. 2020; Liffner et al. 2023).

While the nuclear envelope, the ER, and possibly the IMC (in sporozoites) are likely marked by IMMP, other prominent intracellular membranes like the rhoptries and the plasma membrane appear not to be marked. Similarly, we did not find any evidence indicating that IMMP localized to parasite‐derived membranes in the infected RBCs. Thus, additional experimental work is needed to characterize the specific membrane compartments marked by IMMP. These experiments may also shed light on why mammalian cells did not express IMMP.

For *Plasmodium* spp., IMMP and BODIPY might complement each other in fixed and short‐term live microscopy, highlighting different aspects of parasite membrane composition and architecture. Especially in *Plasmodium* oocysts—parasite stages that, due to their thick proteinaceous cyst wall, are notoriously difficult to stain with external dyes—IMMP revealed a previously underappreciated extensive network of internal membranes. This network encompasses likely nuclear membranes and the ER, but possibly includes other organelles, too.

Notably, IMMP expression in oocysts implied that the DNA occupies only a fraction of the nuclear volume. A DNA‐free region has been previously described for the nuclei of asexual blood stages (Simon et al. 2021) and, indeed, an intranuclear compartmentalization may be a means to organize the genomes in the polyploid nuclei of oocysts. Yet, additional experiments are needed to validate this finding, especially since oocysts are often difficult to stain with dyes, such as Hoechst 33342. Together, this exemplifies how the membrane reporter IMMP could assist in elucidating the little‐understood cell biology of early oocyst development.

In conclusion, here we show that IMMP can serve as a marker for fluorescence imaging of internal membranes of the malaria‐causing parasite *Plasmodium*, in particular for long‐term live cell microscopy. IMMP is also compatible with advanced imaging techniques such as ultrastructure expansion microscopy and works across *Plasmodium* species and stages, rendering it a new tool to study membrane dynamics.

## 4 Experimental Procedures

### 4.1 *P. falciparum* Cell Culture and Drug Treatment

*P. falciparum* 3D7 is a laboratory‐adapted strain, derived from the isolate NF54, which originated from a case of airport malaria in the Netherlands (Ponnudurai et al. 1981). *P. falciparum* 3D7 was cultivated as previously described (Trager and Jensen 1976). In brief, we used fresh O, Rh+ RBCs at 2%–4% hematocrit in supplemented RPMI 1640 medium [with 0.2 mM hypoxanthine (CCPro), 25 mM HEPES, pH 7.4 (Merck), and 12.5 μg/mL gentamicin (Carl Roth), as well as 0.5% AlbuMAX II (gibco)], at 37°C in 90% relative humidity, 5% O_2_, and 3% CO_2_. Parasitemia was monitored via blood smears that were fixed with 100% methanol for 10 s and stained for 1 s in Hemacolor solution II (VWR) and subsequently for 20 s in Hemacolor solution III (VWR) to visualize RBCs and parasites using either a Nikon Eclipse E100 or a Zeiss Axiostar Plus microscope with a 100× oil immersion objective. Routine synchronizations were performed by 5% sorbitol treatment as previously described (Lambros and Vanderberg 1979).

To inhibit the secretory pathway, cells were treated with 5 μg/mL brefeldin A (Sigma‐Aldrich) for 6 h before chemical fixation (see below) and compared to a solvent‐treated control (ethanol) (Benting et al. 1994; Tonkin et al. 2006).

### 4.2 Cloning of Plasmids for *Plasmodium* Transfection

To investigate the 32‐amino acid predicted *P. falciparum* PCNA1 NLS1/IMMP peptide in *P. falciparum*, we expressed IMMP with an additional start codon and fused to GFP using a PR(GGGGS)_2_ linker (with PR deriving from an AvrII site used for molecular cloning) from the episomal plasmid pARL under the control of the *P. falciparum* CRT promoter and the *P. berghei* DHFR/TS terminator (pARL_NLS1::GFP). Additionally, this plasmid contained a WR99210 resistance cassette (Fidock and Wellems 1997). The resulting parasite line was used for fixed and live cell imaging. For Ultrastructure Expansion Microscopy (U‐ExM), we fused IMMP to a HaloTag in the pARL plasmid backbone. All plasmids were sequenced by Sanger sequencing before transfection.

To express IMMP in *P. berghei*, the plasmid pBAT‐SIL6‐MCS (Kooij et al. 2012) was digested with BamHI and NdeI. The putative NLS sequence of *P. falciparum* PCNA1 was amplified from pARL_NLS1::GFP and mScarlet was amplified from the Addgene Plasmid 85054. Fragments were combined using Gibson assembly using the NEB HiFi Assembly kit (NEB). All plasmids were sequenced by Sanger sequencing before transfection. All primers and plasmids used to generate these cell lines are available upon request.

### 4.3 Transfection of *P. falciparum*

Transgenic parasites were generated as previously described (Rug and Maier 2012). In brief, 75–100 μg purified plasmid DNA was resuspended in 30 μL of TE buffer [10 mM tris (pH 8.0) and 1 mM EDTA (pH 8.0)] and added to 370 μL of CytoMix [120 mM KCl, 0.15 M CaCl_2_, 2 mM EGTA, 5 mM MgCl_2_, 10 mM K_2_HPO_4_/KH_2_PO_4_, and 25 mM Hepes (pH 7.6)]. The DNA/CytoMix solution was added to 200 μL packed RBCs infected with ring‐stage *P. falciparum* 3D7 parasites at a parasitemia of 5%–8%. Electroporation was performed in a 0.2 cm gap electroporation cuvette (Bio‐Rad) with a Gene Pulser II (Bio‐Rad, settings: 0.31 kV, 0.950 μF, capacitance set to “High Cap,” resistance on the Pulse Controller II set to “Infinite”). Immediately after electroporation the sample was added to a 10 mL petri dish and cultured under standard conditions. To select for transgenic parasites, we used 2.5 nM WR99210 (kind gift of Jacobus Pharmaceutical Company, Princeton, USA).

### 4.4 *P. falciparum* Preparation for Fixed‐Cell Imaging

Fluorescence staining for microscopy of chemically fixed blood‐stage parasites was done as previously described with minor alterations (Simon et al. 2021; Machado et al. 2023). In brief, we seeded cells on ibidi ibiTreat 8‐well dishes, rinsed them with pre‐warmed PBS and subsequently fixed with pre‐warmed 4% PFA/PBS for 20 min at 37°C. After fixation, cells were washed with PBS and permeabilized with 0.1% Triton X‐100/PBS for 15 min at room temperature (RT). Cells were then washed two times with PBS, leaving 200 μL of PBS in each well for imaging. Additionally, Hoechst 33342 was added approximately 1 h before imaging to label the nuclei, while BODIPY TR Ceramide (Thermo Fisher Scientific, D7540) was added 20 min prior to imaging to visualize membranes. Endogenous GFP signal was strong enough to not require additional antibody staining before imaging.

### 4.5 *P. falciparum* Preparation for Live‐Cell Imaging

For live‐cell imaging, infected RBCs were seeded in either a round 35 mm imaging dish or an ibidi 8‐well dish. When using ibidi ibiTreat‐coated dishes, the dishes were rinsed twice with PBS before seeding. Uncoated dishes were coated prior to seeding with concanavalin A (200 μL for round dish; 80 μL/well for an 8‐well dish) and incubated at 37°C for 20–30 min. Excess concanavalin A was removed, and dishes were rinsed with PBS twice.

To prepare the cells, 500 μL of resuspended cell culture was pelleted (800 g for 2 min) and washed twice with supplemented RPMI 1640 without AlbuMAX II. Cells were resuspended in 1 mL of pre‐warmed supplemented RPMI 1640 without AlbuMAX II, and 125 μL per 8‐well or 250 μL per 35 mm round dish was used for seeding. To allow for cell attachment, dishes were incubated for 10 min at 37°C. To create a monolayer, the cells were gently washed and kept under normal culture conditions in supplemented RPMI 1640 without AlbuMAX II until the medium was changed to pre‐warmed imaging medium [i.e., supplemented RPMI 1640 without phenol red (PAN‐Biotech)]. The SPY650‐DNA dye was added to the imaging medium 2 h prior to imaging and BODIPY TR Ceramide was added 20 min prior to imaging.

### 4.6 *P. falciparum* Preparation for Ultrastructure‐Expansion Microscopy (U‐ExM)

Ultrastructure Expansion Microscopy was performed as previously described (Gambarotto et al. 2019; Bertiaux et al. 2021; Liffner and Absalon 2021). In brief, 12 mm coverslips were coated with poly‐D‐Lysin for 1 h at 37°C and then washed three times with PBS. PBS was removed and coverslips were stored at 4°C in a humid chamber. *P. falciparum*‐infected RBCs were enriched by magnetic affinity purification using a MACS LS column (Miltenyi Biotec), the eluate was pelleted (800 g for 2 min) and the supernatant was removed. Cells were resuspended in 300 μL supplemented RPMI 1640 without AlbuMAX II and 100 μL of the suspension were added to the coverslip. After the seeding for 10 min at 37°C, the supernatant was removed and the infected RBCs were chemically fixed by adding 50 μL of 4% PFA/PBS followed by 20 min incubation at 37°C. Then, coverslips were washed with PBS and stored in a 12‐well plate in PBS. To later permit protein anchoring to the poly acrylamide gel, we added 1 mL formaldehyde acrylamide (FA/AA) solution to the cells and incubated at 37°C for 3.5–5 h with gentle agitation on an orbital shaker plate at 80 rpm.

Gelation was carried out on ice in a humid chamber by dipping the coverslips in PBS and then placing them on a 35 μL drop of monomer solution with tetramethylethylendiamine (TMED, Thermo Fisher Scientific) and ammonium persulfate (APS, Thermo Fisher Scientific) with cells facing the monomer solution mixture. After incubation for 5 min on ice, the humid chamber was covered with aluminum foil and incubated for 1 h at 37°C. Coverslips were transferred with the gel facing up in a 6‐well plate containing 2 mL denaturation buffer and were incubated until the gel detached from the coverslip. Then, gels were transferred into a 1.5 mL reaction tube with fresh denaturation buffer and kept for 1.5 h at 95°C. Denaturation buffer was removed, and gels were left for the first expansion in ddH_2_O. After 30 min, the water was replaced by fresh water supplemented with sodium acetate and left overnight at RT. Next, the gel size was measured to determine the expansion factor. For immunofluorescence staining, the gels were cut and incubated in PBS twice for 15 min, which leads to shrinkage of the gels. PBS was replaced by 3% BSA/PBS and incubated for 30 min at RT, then the primary antibody was added (250 μL, 1:1000 anti‐halo, Promega) and incubated for 3 days at 4°C with gentle agitation on an orbital shaker plate. Next, the gels were washed five times for 10 min with 0.5% Tween/PBS and the secondary antibody solution (anti‐mouse Alexa Fluor 488, 1:500, Invitrogen) with Hoechst 33342 (1:100 Thermo Fischer Scientific) was added and incubated for 2.5 h in the dark with gentle agitation at 37°C. After five washes with 0.5% Tween/PBS for 10 min each, Atto 647 NHS ester (Atto‐Tech) staining was performed for 1.5 h at RT with gentle agitation. After washing as described above, the gels were placed in water supplemented with BODIPY TR Ceramide (2 μM) for the second expansion and for membrane staining. For imaging, Ibidi Glass bottom dishes were coated with poly‐D‐Lysine and gels were placed in the wells with cells facing downwards.

### 4.7 Expression and Localization of IMMP::GFP in Mammalian Cells

HeLa‐derived TZM‐bl cells (Wei et al. 2002) were transfected with GFP or IMMP::GFP expression constructs using Lipofectamine 2000 (Thermo Fisher Scientific) according to the manufacturer's protocol. In brief, 5 × 10^4^ TZM‐bl cells were seeded on glass coverslips overnight and then transfected using 1 μg of DNA and 2 μL of lipofectamine. Samples were then harvested 48 h post transfection, the cells were washed in 1 × PBS, and the plasma membrane was stained using WGA‐647 (Invitrogen) at a final concentration of 1 μg/mL for 5 min at RT. The samples were then fixed using 3% PFA for 15 min in the dark and further processed for light microscopy analysis. Point laser scanning confocal microscopy was then performed on a Leica SP8 microscope using an HC PL APO CS2 63 ×/1.4 N.A. oil immersion objective. Images were acquired using PMT detectors in reflection mode with laser excitation at 488 or 633 nm, and spectral detection windows set between 500 to 570 and 650 to 700 nm wavelengths.

Western blot analysis was done according to standard procedures. In brief, TZM‐bl were lysed with 2 × SDS sample buffer (10% glycerol, 6% SDS, 130 mM Tris HCl pH 6.8, 10% *β*‐mercaptoethanol) supplemented with protease/phosphatase inhibitor (Thermo Fisher Scientific, 78440) and boiled for 5 min at 95°C. Protein samples were resolved by SDS‐PAGE electrophoresis and blotted onto nitrocellulose membranes. Membranes were blocked in 5% milk/PBST for 1 h and were incubated with primary antibodies overnight at 4°C (rat anti‐GFP: 1:1000, Chromotek, 3H9; mouse anti‐GAPDH: 1:5000, Biorad, MCA4740). After three washes with 5% milk/PBST, membranes were incubated with HRP‐conjugated secondary antibodies (Goat anti‐mouse IgG‐HRP: 1:5000, 31,431; rabbit anti‐rat IgG‐HRP: 1:5000, PA1‐28786; Invitrogen) for 1 h at room temperature. Membranes were again washed three times and visualized by chemiluminescence.

### 4.8 Ethics Statement and Mice

All experiments were performed in accordance with GV‐SOLAS and FELASA guidelines and have been approved by the German authorities (Regierungspräsidium Karlsruhe). Female Swiss mice weighing 20–25 g were purchased from JANVIER and were aged 5–10 weeks at the time point of infection. For each experiment, mice were age‐matched and were allocated randomly to each group. Mice were kept in groups of 2–4 mice per cage under specified pathogen‐free conditions within the animal facility at Heidelberg University on a 12‐h light/dark cycle at 22°C (±2°C) and 50%–60% humidity with *ad libitum* access to food and water.

### 4.9 General Maintenance of Parasites and Mosquitoes

Mice were typically infected with *P. berghei* by intraperitoneal injection of cryostabilates, consisting of 100 μL parasitized blood and 200 μL freezing solution (Alsever solution plus 10% glycerol), or by intraperitoneal or intravenous fresh blood transfer of a defined number of parasites. Parasitemia was determined using Giemsa‐stained blood smears. Parasitized blood was collected from infected mice by cardiac puncture of fully anesthetized mice (after intraperitoneal application of ketamine/xylazine). This was also the route to euthanize the mice. Mosquitoes (*Anopheles stephensi*) were reared and maintained according to established protocols.

### 4.10 Generation of Transgenic *P. berghei* Parasites

Transgenic *P. berghei* parasite lines were generated largely as previously described (Janse et al. 2006; Schwach et al. 2015). Briefly, 10 μg plasmid DNA was digested over night with PvuI, precipitated using ethanol and resuspended in 10 μL PBS. Schizonts were obtained by culturing 500 μL blood containing > 1% parasitemia in schizont medium (RPMI‐1640, gibco) supplemented with 20% FCS (gibco) and 1 μg/mL gentamycin (PAA) for about 20 h at 37°C and were purified over a 55% Nycodenz (Axis‐shield diagnostics) gradient. DNA and schizonts were mixed with 100 μL Nucleofector solution from the human T cell Nucleofector Kit, electroporated using the Amaxa Nucleofector II device (Lonza), and immediately injected intravenously into a mouse. Transgenic parasites were selected by administering pyrimethamine (7 μg/mL, Sigma‐Aldrich) to the drinking water 1 day after transfection. Blood‐stage positive mice were bled by cardiac puncture and blood was used for preparation of cryostabilates and for genotyping. To this end, blood containing at least 1% parasitemia was lysed using 0.093% saponin and the parasite pellet was resuspended in 200 μL PBS. gDNA was isolated using the DNeasy Blood & Tissue Kit (Qiagen) according to the manufacturer's instructions. Parasites were genotyped by PCR for amplification of the wildtype locus (locus), 5′ integration (5′ int), and 3′ integration (3′ int) sites. No clonal lines were generated. Instead, mice were continuously kept on pyrimethamine to maintain selection pressure. For all microscopy, IMMP::mScarlet‐expressing parasites were identified based on their strong red fluorescence and transfection efficacy was determined to be above 90%.

### 4.11 Determination of *P. berghei* Asexual Growth Rate

To determine parasite growth, three mice per line were infected intravenously with 1000 infected RBCs. Parasitemia was assessed from Day 4 to Day 7 post infection by Giemsa‐stained blood smears. For each mouse, the parasite growth rate was determined from the slope of a linear regression fitted to the log‐transformed parasitemia curve (excluding values for Day 7, where parasitemia plateaued).

### 4.12 Live and Fixed‐Cell Imaging of Asexual *P. berghei* Parasites

To perform life‐cell imaging, a drop of *P. berghei* parasitized mouse blood collected from the tail vein was stained with BODIPY FL C5‐Ceramide (Thermo Fischer Scientific, D3521, 1:1000 dilution) for 1 h at 37°C and with Hoechst 33342 (final concentration 10 μg/mL) for 5 min at 37°C, washed once, and imaged on an inverted Zeiss LSM900 microscope equipped with an Airyscan 2 detector (see below).

To fix cells for imaging, 50 μL of blood was mixed with 200 μL of RPMI medium, and immediately fixed by adding an equal volume of 4% PFA/0.0075% glutaraldehyde in PBS. Cells were incubated for 30 min at 37°C, washed once in PBS and stored in 3% BSA/PBS at 4°C. Prior to imaging, cells were stained with BODIPY FL (1:1000 dilution) for 1 h at RT and with Hoechst 33342 (final concentration 10 μg/mL) for 5 min at RT, washed once and imaged.

### 4.13 Ookinete Culture

To obtain ookinetes for live‐cell imaging, mice were infected with 2 × 10^7^ iRBC i.p. Three days later, mice were bled, and the gametocyte‐containing blood transferred to 10 mL ookinete medium (RPMI supplemented with 20% (v/v) FCS, 50 μg/mL hypoxanthine, and 100 μM xanthurenic acid, pH 7.8–8.0). Parasites were cultured at 19°C for 20–24 h, stained for 1 h with BODIPY FL (1:1000 dilution) and for 5 min with Hoechst 33342 (final concentration 10 μg/mL) at 19°C, washed once in ookinete medium and immediately imaged.

### 4.14 Mosquito Infections

Mosquitoes were infected by blood meal on a mouse 3 days post infection with 2 × 10^7^ iRBC i.p. To this end, the mouse was anesthetized by administering ketamine/xylazine solution (20 mg/mL ketamine, 0.6 mg/mL xylazine in PBS) i.p. at 5 μL/g body weight and placed onto a mosquito cage containing approximately 150–250 mosquitoes. Mosquitoes were allowed to feed for approximately 30 min, with a change of mouse position after 15 min. After feeding, mosquitoes were immediately kept at 21°C and 80% humidity, and fed with 10% (v/v) saccharose with 0.05% PABA and 1% (v/v) NaCl.

At selected time points, mosquito midguts were dissected into PBS, stained with BODIPY FL (1:1000 dilution) and Hoechst 33342 (final concentration 10 μg/mL) for 1 h at RT, washed once, and imaged. For oocysts on Day 4, only staining with Hoechst 33342 was performed.

### 4.15 Imaging at a Zeiss LSM900 Microscope

For all imaging, unless stated otherwise, point laser scanning confocal microscopy was performed on an inverted Zeiss LSM900 microscope equipped with an Airyscan 2 detector using a Plan‐Apochromat 63×/1,4 oil immersion objective and with the Airyscan detector in SR mode.

For short‐term imaging of live and fixed cells, single z‐stacks were recorded to cover the entire cell, ranging from 11 to 159 confocal slices. Long‐term live‐cell imaging was performed at 37°C with 5% CO_2_ and 5% O_2_, and 11 confocal slices per z‐stack were recorded every 15 min over approximately 14 h. Multiple positions were imaged using the “Positions” tool and the focus was stabilized using the “Definite Focus” mode. Multichannel images were acquired sequentially using the 405 nm diode for Hoechst 33342, the 488 nm laser for GFP or BODIPY FL, the 561 nm diode for SPY555‐DNA, BODIPY TR Ceramide or mScarlet, and the 640 nm laser for SPY650‐DNA. The scanning was done bidirectionally and the transmitted light images were captured by the T‐PMT detector. Airyscan data was processed with the ZEN Blue 3.1. software.

### 4.16 Image Analysis

Image analysis was performed using Fiji (Schindelin et al. 2012). For snapshots of a single time point (fixed cells, live‐cell snapshots, and U‐ExM), a single z‐slice at the center of the cell was selected for analysis and visualization. Channels were converted to gray scale, and inverted to display a black signal on a white background for enhanced visibility. To compare the signal intensities of BODIPY TR Ceramide and IMMP::GFP, we used line profiles. Lines were drawn from the extracellular space or cytosol, across the membrane, and into the cytoplasm or nucleus, respectively. Signal intensities along these lines were measured using the plot profile function of Fiji. Intensities were min‐max normalized and the maximal BODIPY TR Ceramide value was set at the distance 0 μm for graphical representation. Long‐term imaging data was pre‐processed to correct for drift, using the 3D Drift Correction in Fiji. To quantitatively assess the GFP signal from long‐term imaging data, a region of interest was first cropped out. Then the sum of the signal (i.e., the raw integrated density) of all z‐slices per time point was plotted over time in arbitrary units. For Kaplan–Meier plots, long‐term imaging data was used to evaluate cell survival over time. Time of cell death was determined based on loss of structural integrity, that is, cell lysis of parasite and/or RBC. Further data processing and visualization were performed using Microsoft Office, Python (Jupyter notebook), and GraphPad Prism.

## Author Contributions

**Leah Zink:** conceptualization, methodology, investigation, writing – original draft, writing – review and editing. **Viola Reuschenbach:** investigation. **Samy Sid Ahmed:** investigation, resources, writing – review and editing. **Jana Niethammer:** investigation. **Muriel Lauer:** investigation. **Markus Ganter:** conceptualization, methodology, writing – original draft, writing – review and editing, visualization, supervision, funding acquisition. **Oliver T. Fackler:** writing – review and editing, funding acquisition. **Franziska Hentzschel:** conceptualization, methodology, writing – original draft, visualization, supervision, funding acquisition, writing – review and editing. **Katharina Zorko:** resources. **Aistė Kudulytė:** investigation.

## Funding

F.H. receives funding from the ERC (Starting Grant PlasmoArp, 101162759) and the German Research Foundation (Deutsche Forschungsgemeinschaft, DFG) through SPP 2225, project number 531930468. M.G. is supported by the German Research Foundation (Deutsche Forschungsgemeinschaft, DFG), project number 572009475; the Advanced Medical Scientist Program of Heidelberg University, Faculty of Medicine; the Health + Life Science Alliance Heidelberg Mannheim, receiving state funding approved by the State Parliament of Baden‐Württemberg. O.T.F. and M.G. receive support from the German Research Foundation (Deutsche Forschungsgemeinschaft, DFG) through SFB 1129, project number 240245660, subprojects TP8 and TP18, respectively.

## Conflicts of Interest

The authors declare no conflicts of interest.

## Supporting information


**Figure S1:** BODIPY TR ceramide staining affects RBC survival during live‐cell imaging. (A) Survival of uninfected RBCs during imaging. (B) Survival of *P. falciparum*‐infected RBCs during imaging. (A, B) Transmission images were acquired with 0.1% laser power.
**Figure S2:** AlphaFold3 predicts IMMP to be an amphiphilic peptide that harbors the membrane targeting activity. (A) AlphaFold3 prediction of IMMP (cyan) fused to GFP via a linker peptide (PR[GGGGS]2, with PR deriving from an AvrII site for molecular cloning). (B) Surface rendering of IMMP with hydrophobic and hydrophilic amino acid (AA) residues indicated in yellow and cyan, respectively. (C) Short‐term live‐cell imaging of parasites expressing GFP co‐stained with BODIPY TR Ceramide (left) or the DNA dye SPY650 DNA (right) (one biological replicate). (D) Images of cells expressing IMMP::GFP and treated with brefeldin A (left panel) or a solvent control (right panel) for 6 h before fixation and imaging. Cells were co‐stained with the DNA dye Hoechst 33342 (one biological replicate). Scale bars, 2 μm.
**Figure S3:** Ultrastructure expansion microscopy of IMMP::Halo expressing *P. falciparum*. IMMP::Halo expressing cells were co‐stained with BODIPY TR Ceramide and the DNA dye Hoechst 33342 (top row) or with Hoechst 33342 alone (bottom rows); scale bar, 4 μm.
**Figure S4:** Generation and basic characterization of an IMMP::mScarlet expressing *P. berghei* cell line. (A) Integration strategy to generate the IMMP::mScarlet cell line. The silent locus on chromosome 6 (SIL6) was targeted with a linearized integration construct containing two homology regions (SIL6L, SIL6R), an IMMP::mScarlet expression cassette, as well as a cassette conferring drug resistance (selection cassette). Upon homologous recombination, this construct is integrated in the SIL6 locus. Test primer combinations for diagnostic PCR are indicated by arrow heads and the expected fragments are shown as lines. (B) Diagnostic PCR confirms the successful integration of IMMP::mScarlet into the SIL6 locus. (C) Asexual blood‐stage proliferation of *P. berghei* expressing IMMP::mScarlet was compared to wild type parasites after i.v. injection of 1000 infected RBCs of each cell line into recipient animals (*n* = 3). Parasitemia was determined daily using Giemsa‐stained blood smears. Line, linear regression, used to determine growth rate.
**Figure S5:** IMMP::eGFP transfection in human cells. (A) Control untransfected HeLa‐derived TZM‐bl cells stained with DAPI to detect DNA and wheat germ agglutinin (WGA) to mark the plasma membrane. (B) Control TZM‐bl cells transfected with a GFP expressing construct and staind with DAPI and WGA; inset, highlighting the pancellular localization of GFP (one biological replicate). (C) TZM‐bl cells transfected with a IMMP::GFP expressing construct and staind with DAPI and WGA; inset, highlighting the IMMP::GFP signal akin to the control GFP signal in B (one biological replicate). (D) Western blot analysis of TZM‐bl cells (untransfected, GFP expressing, or IMMP::GFP expressing), with GAPDH serving as a loading control (one biological replicate). Scale bars, 20 μm.

## Data Availability

The data that support the findings of this study are available from the corresponding author upon reasonable request.
