# Supplementary material for: A Genetically Encoded Marker for Imaging of Internal Plasmodium Membranes
Source: Mol Microbiol. 2026 Sep 15;126(4):462–72. doi: 10.1111/mmi.70098 (PMC13644370; doi:10.1111/mmi.70098)
Supplement: Supplementary file 1 [file MMI-126-462-s001.pdf]

Supplementary Information

A genetically encoded marker for imaging of internal *Plasmodium* membranes

Leah Zink [1,#], Viola Reuschenbach [1], Samy Sid Ahmed [2], Muriel Lauer [1], Jana Niethammer [1], Aistė Kudulytė [1], Katharina Zorko [1], Oliver T. Fackler [2,3], Franziska Hentzschel [1], Markus Ganter [1,†]

[1] Center for Infectious Diseases, Parasitology, Medical Faculty Heidelberg, Heidelberg University, Heidelberg, Germany

[2] Center for Infectious Diseases, Integrative Virology, Medical Faculty Heidelberg, Heidelberg University, Heidelberg, Germany

[3] German Centre for Infection Research (DZIF), Partner Site Heidelberg, Heidelberg, Germany

[#] Current address: Centre for Structural Systems Biology, Cell Biology of Human Parasites Group, Hamburg, Germany

[†] Correspondence: markus.ganter@med.uni-heidelberg.de

Supplementary figures

Fig. S1

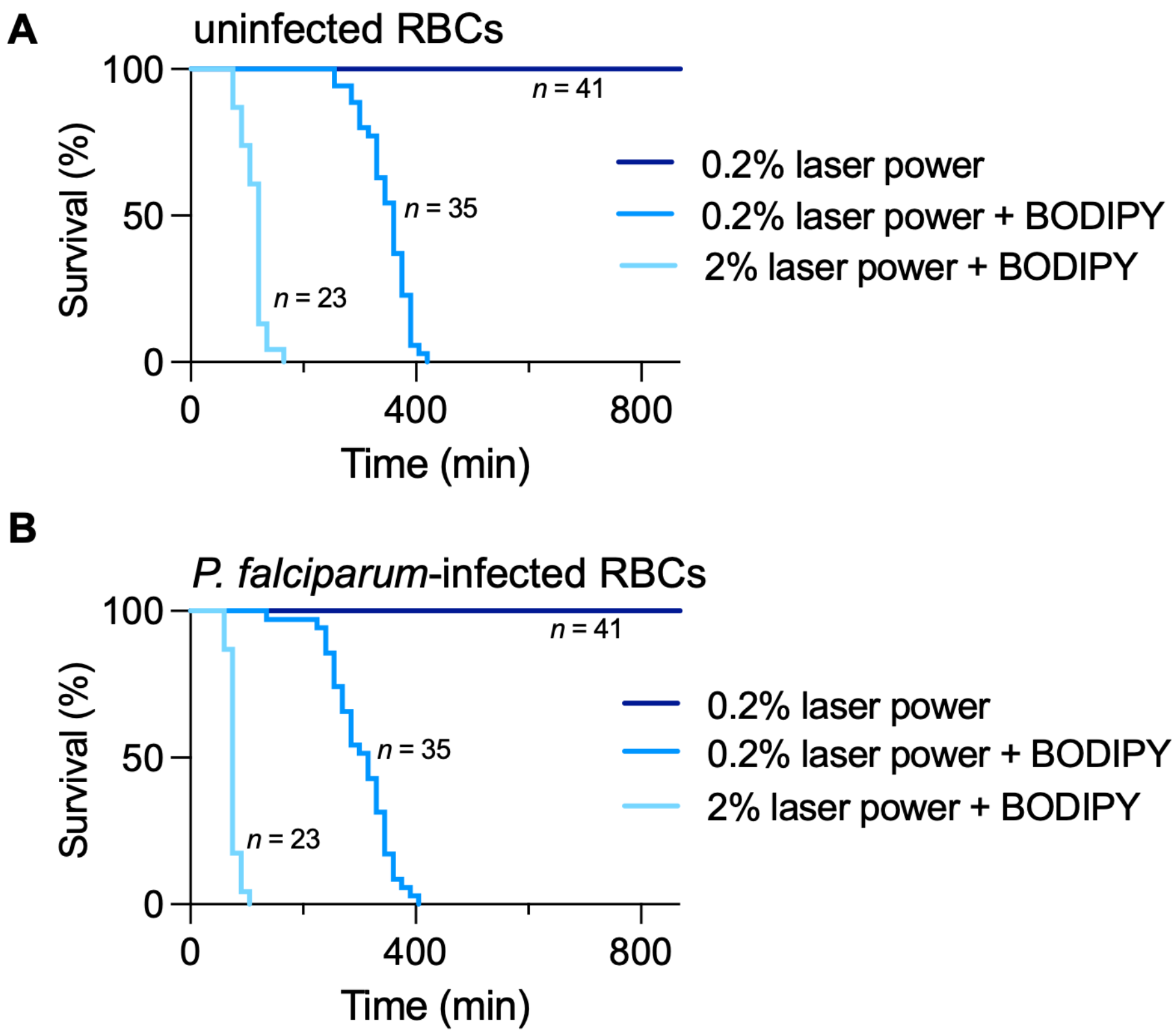

Fig. S1: BODIPY TR Ceramide staining affects RBC survival during live-cell imaging. A Survival of uninfected RBCs during imaging. **B** Survival of *P. falciparum*-infected RBCs during imaging. A, B; Transmission images were acquired with 0.1% laser power.

Fig. S2

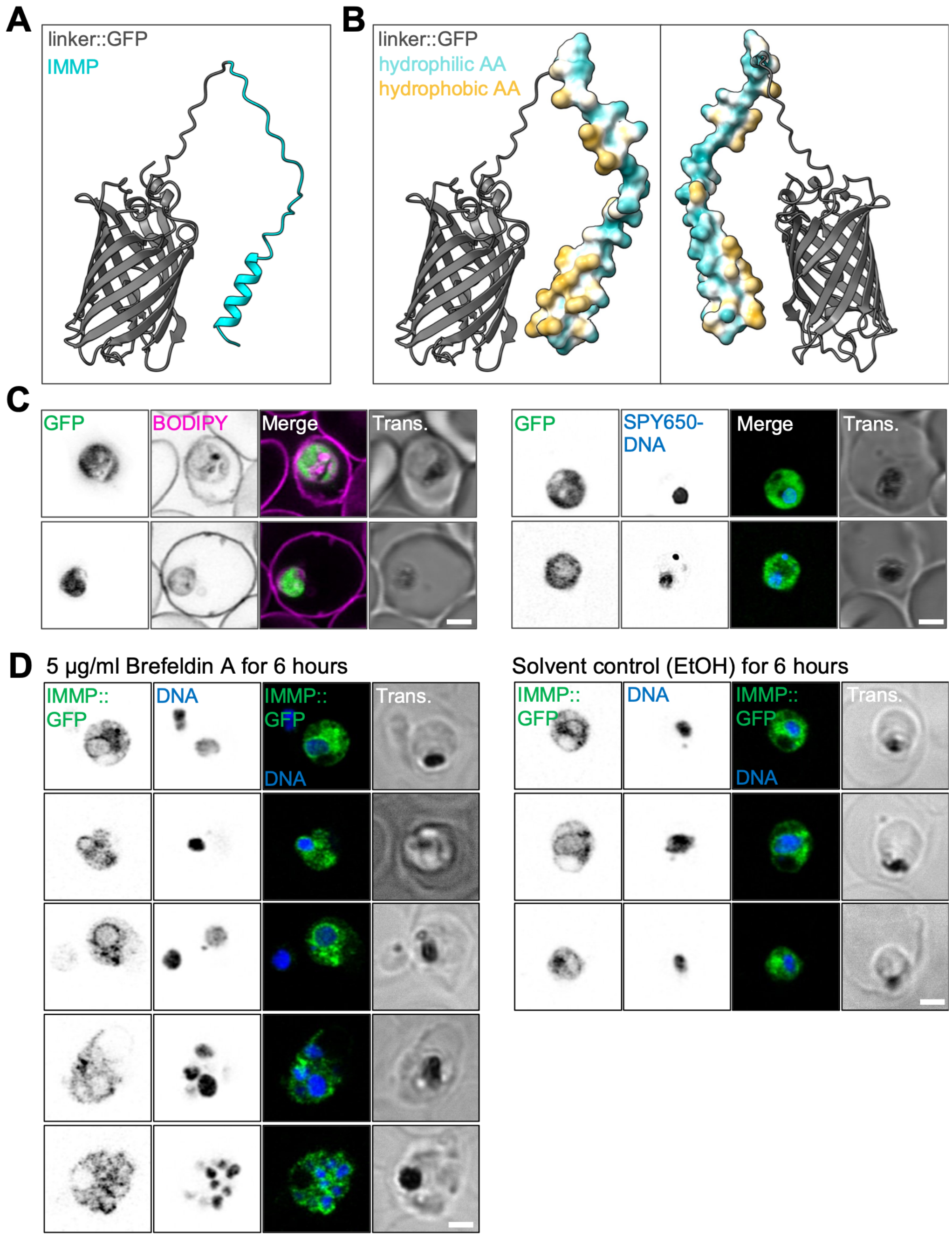

Fig. S2: AlphaFold3 predicts IMMP to be an amphiphilic peptide that harbors the membrane-targeting activity. A AlphaFold3 prediction of IMMP (cyan) fused to GFP via a linker peptide (PR[GGGGS]$_2$, with PR deriving from an AvrII site for molecular cloning). **B** Surface rendering of IMMP with hydrophobic and hydrophilic amino acid (AA) residues indicated in yellow and cyan, respectively. **C** Short-term live-cell imaging of parasites expressing GFP co-stained with BODIPY TR Ceramide (left) or the DNA dye SPY650-DNA (right) (one biological replicate). **D** Images of cells expressing IMMP::GFP and treated with brefeldin A (left panel) or a solvent control (right panel) for 6 hours before fixation and imaging. Cells were co-stained with the DNA dye Hoechst 33342 (one biological replicate). Scale bars, 2 µm.

Fig. S3

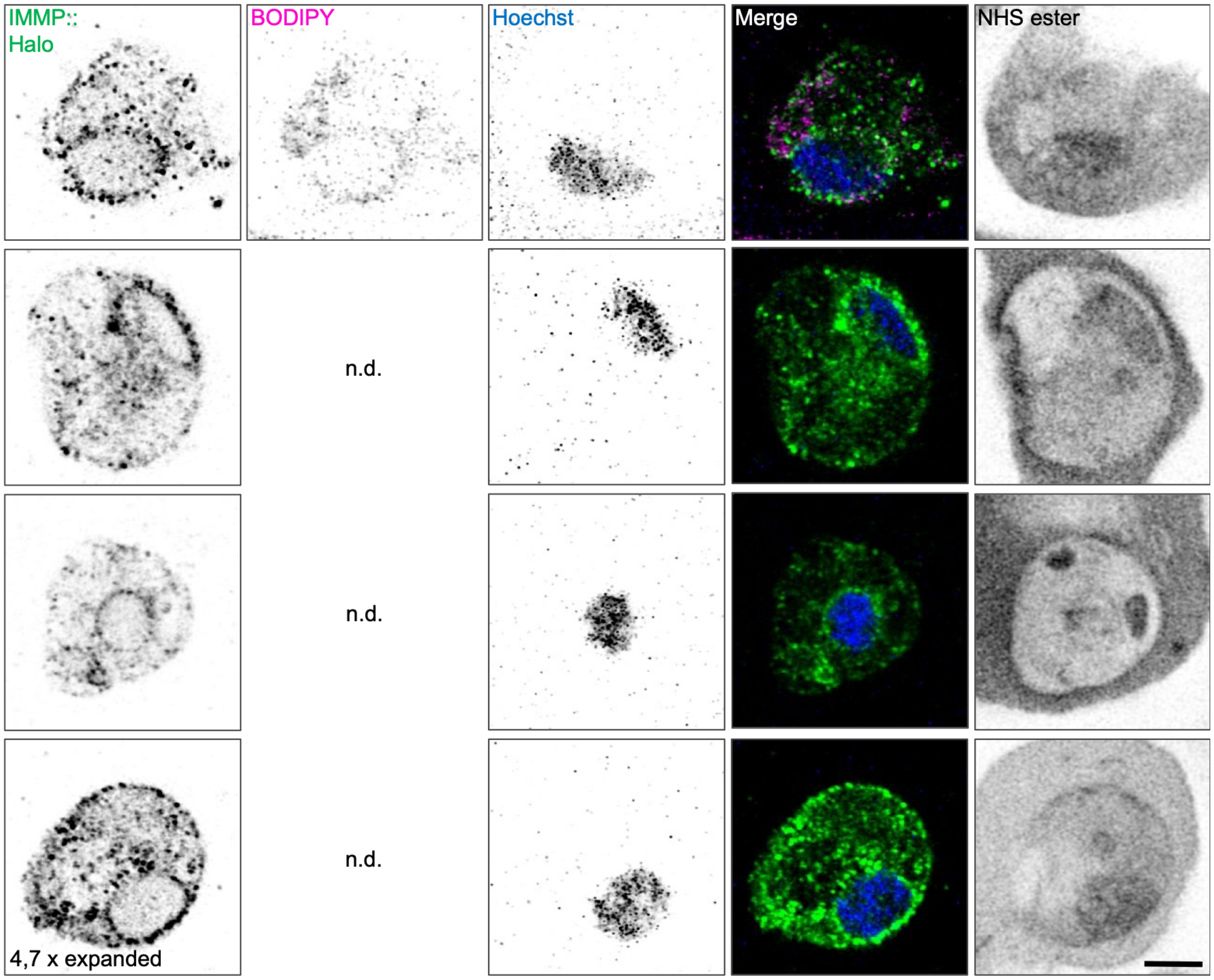

Fig. S3: Ultrastructure expansion microscopy of IMMP::Halo expressing *P. falciparum*. IMMP::Halo expressing cells were co-stained with BODIPY TR Ceramide and the DNA dye Hoechst 33342 (top row) or with Hoechst 33342 alone (bottom rows); scale bar, 4 µm.

Fig. S4

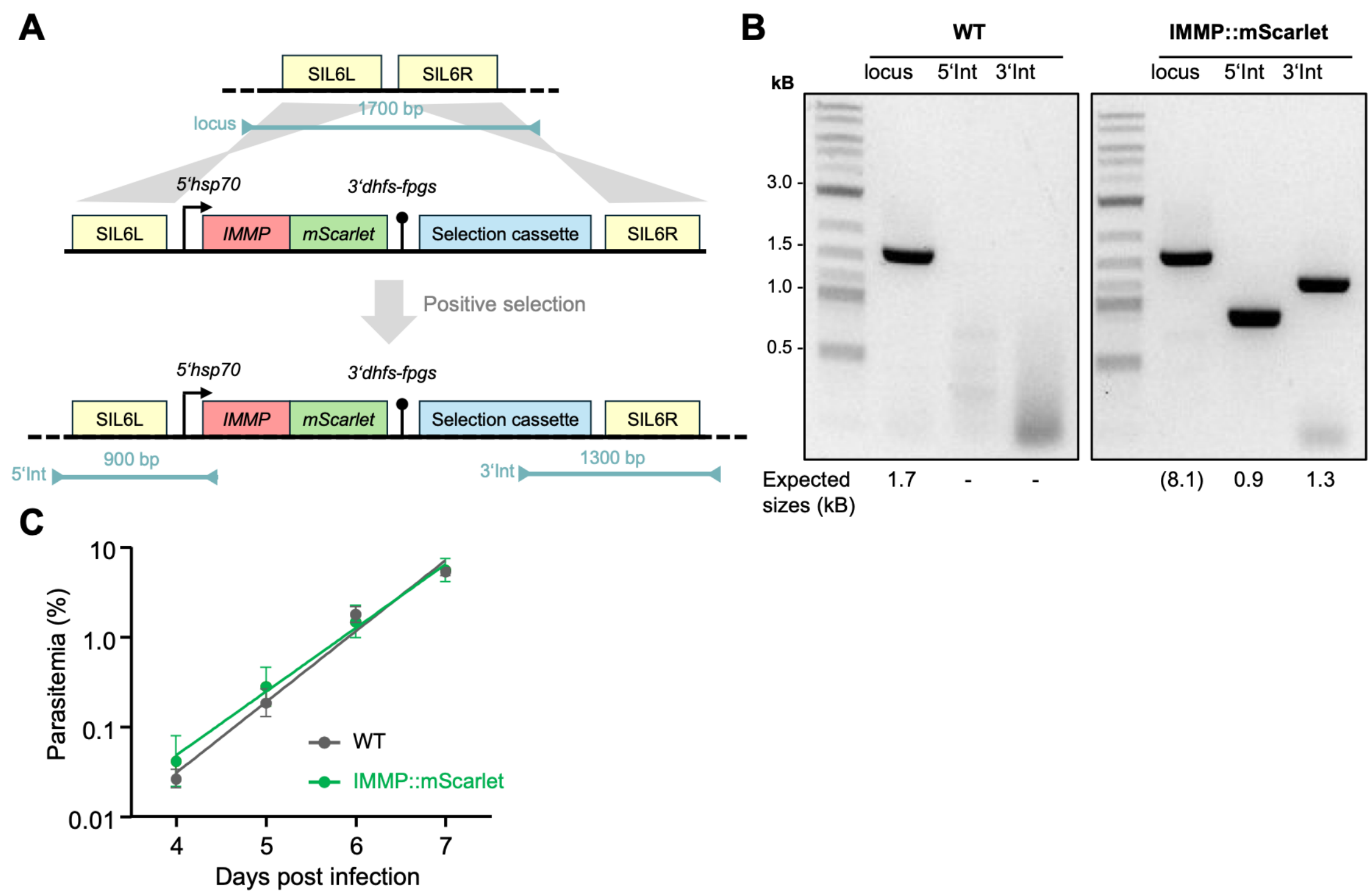

Fig. S4: Generation and basic characterization of an IMMP::mScarlet expressing *P. berghei* cell line. A Integration strategy to generate the IMMP::mScarlet cell line. The silent locus on chromosome 6 (SIL6) was targeted with a linearized integration construct containing two homology regions (SIL6L, SIL6R), an IMMP::mScarlet expression cassette, as well as a cassette conferring drug resistance (selection cassette). Upon homologous recombination, this construct is integrated in the SIL6 locus. Test primer combinations for diagnostic PCR are indicated by arrow heads and the expected fragments are shown as lines. **B** Diagnostic PCR confirms the successful integration of IMMP::mScarlet into the SIL6 locus. **C** Asexual blood-stage proliferation of *P. berghei* expressing IMMP::mScarlet was compared to wild type parasites after i.v. injection of 1000 infected RBCs of each cell line into recipient animals ($n = 3$). Parasitemia was determined daily using Giemsa-stained blood smears. Line, linear regression, used to determine growth rate.

Fig. S5

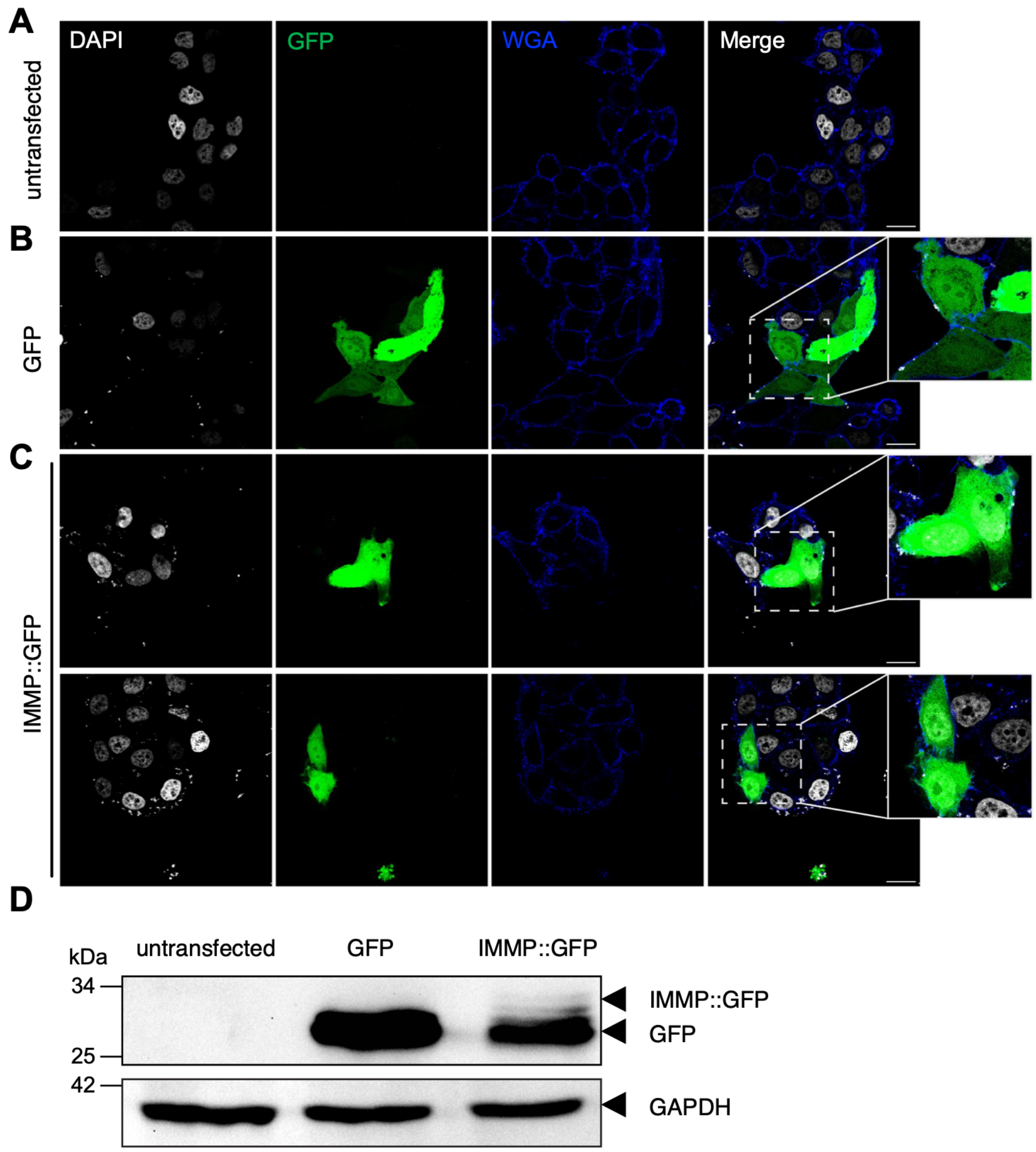

Fig. S5: IMMP::eGFP transfection in human cells. A Control untransfected HeLa-derived TZM-bl cells stained with DAPI to detect DNA and wheat germ agglutinin (WGA) to mark the plasma membrane. **B** Control TZM-bl cells transfected with a GFP expressing construct and staind with DAPI and WGA; inset, highlighting the pancellular localization of GFP (one biological replicate). **C** TZM-bl cells transfected with a IMMP::GFP expressing construct and staind with DAPI and WGA; inset, highlighting the IMMP::GFP signal akin to the control GFP signal in B (one biological replicate). **D** Western Blot analysis of TZM-bl cells (untransfected, GFP expressing, or IMMP::GFP expressing), with GAPDH serving as a loading control (one biological replicate). Scale bars, 20 µm.
